# Pan-Cancer Pre-Treatment ctDNA Detection Using a Highly Sensitive Tumor-Informed Assay †

**DOI:** 10.3390/ijms27093979

**Published:** 2026-04-29

**Authors:** Scott Strum, Clodagh Murray, Sofia Genta, Enrique Sanz-Garcia, Albiruni Razak, Stephanie Lheureux, Christodoulos Pipinikas, Ben X. Wang, Amber Chevalier, Christopher G. Smith, Anna Spreafico, Lillian L. Siu, Mitchell Elliott, David W. Cescon

**Affiliations:** 1 Department of Medical Oncology and Hematology, Princess Margaret Cancer Centre, University Health Network, Toronto, ON M5G 2M9, Canada; 2 Department of Medical Oncology, Arthur J.E. Child Comprehensive Cancer Centre, University of Calgary, Calgary, AB T2N 5G2, Canada; 3 NeoGenomics, Inc., Fort Myers, FL 33912, USA; 4 Department of Oncology, Queen’s School of Medicine, Queen’s University, Kingston, ON K7L 3N6, Canada

**Keywords:** ctDNA, MRD, cancer, biomarkers

## Abstract

Circulating tumor DNA (ctDNA) has many potential applications in the management of cancer, including detection, monitoring, and treatment response assessment. This study evaluates pre-treatment ctDNA detection in a single-institution pan-cancer cohort using a highly sensitive tumor-informed ctDNA assay. Participant samples were collected at the University Health Network across six study cohorts. A total of 273 patients with stage II-IV cancers spanning 16 tumor types were analyzed using a personalized tumor-informed assay (RaDaR^®^). Harmonized methods were employed to mitigate the impact of variables known to influence ctDNA detection and its quantification. Pre-treatment ctDNA was detected in 83% of participants (226/273) across all stages and cancer types. Detection rates varied by clinical characteristics, including cancer type and stage; ctDNA was detected in 100% of stage IV metastatic recurrent high-grade serous ovarian cancer (HGSOC) patients (n = 8/8) and 54% of stage IV melanoma patients (n = 6/11). The median estimated variant allele fraction (eVAF) across all patients with a positive ctDNA result was 0.25% (range: 0.000029–37.7%), varied by tumor type and increased with cancer stage. A total of 11% of positive samples (24/226) had an eVAF of <0.01%. This pan-cancer ctDNA analysis using a highly sensitive assay reveals high detection rates overall, including ~10% at low levels (<0.01% eVAF), offering a reference for future clinical ctDNA applications and furthering our understanding of factors influencing ctDNA measurements.

## 1. Introduction

Cancer biomarkers have an important role in modern oncology care and research [1,2]. They hold established applications across a number of clinical contexts, including screening, diagnosis, prognostication, treatment selection, and monitoring. While blood-based tumor markers have a role in clinical management, they are limited in many cases by their sensitivity and/or specificity. An emerging class of blood-based biomarkers, which detect and measure circulating tumor DNA (ctDNA), may offer improved test characteristics and could improve the management of patients with both early- and advanced-stage cancers.

ctDNA is a tumor-derived subset of cell-free DNA (cfDNA) found in the blood circulation. DNA from tumors can also be detected in other body fluids, including cerebrospinal fluid, saliva, and urine [3]. Clinically, detection rates and levels of ctDNA are associated with tumor burden [4,5], anatomic location of disease [6], and changes in response to treatments such as chemotherapy, targeted therapy, immunotherapy, radiation therapy, and surgery [7,8,9,10]. For example, in the iSPY-2 trial, ctDNA detection showed strong predictive and prognostic value in patients undergoing neoadjuvant treatment for high-risk early-stage breast cancer [11]. In that setting, a lack of ctDNA clearance prior to surgery was associated with metastatic recurrence. While validation of the clinical utility of ctDNA assays in disease management is ongoing, some interventional clinical trials have shown promising applications, such as in the ctDNA-guided adjuvant treatment of patients with resected stage II colon cancer [12].

Technological advances have resulted in improved assays with more sensitive ctDNA detection [13]. In general, ctDNA detection assays use either a tumor-informed or tumor-agnostic approach [14,15,16,17]. Tumor-informed methods rely on sequencing a patient’s matched tumor tissue to identify tumor-specific somatic alterations, allowing design of assays for sensitive ctDNA detection, while minimizing false-positive, non-tumor-derived signals such as clonal hematopoiesis of indeterminate potential (CHIP) [16,17]. However, the use of tumor tissue adds complexity to the analysis workflow and may increase turnaround times. By contrast, tumor-agnostic ctDNA assays do not rely on prior tissue analysis and instead evaluate common cancer-associated mutations, genomic alterations, or methylation patterns. Although more convenient, tumor-agnostic methods may have lower sensitivity or specificity, and some approaches may be more susceptible to false positives from non-tumor sources like CHIP.

Development of ctDNA assays typically involves the characterization of analytical sensitivity and specificity in patient-derived samples. As the development of strategies employing ctDNA in the clinical setting continues, including through interventional trials (e.g., [12]), an understanding of ctDNA detection rates across tumor types and disease stages is essential. Relatively few pan-cancer ctDNA studies have been published to date [18,19]. The aim of this study was to evaluate pre-treatment ctDNA detection rates and levels in a single-institution pan-cancer cohort using RaDaR, a tumor-informed ctDNA assay. Harmonizing the methodology and pre-treatment timing of blood collection was intended to minimize the impact of variables known to influence ctDNA levels. Similarly, the use of the same assay across all samples limits the variability between assays that differ in approach, analysis, and interpretation.

## 2. Results

**Figure 1:**
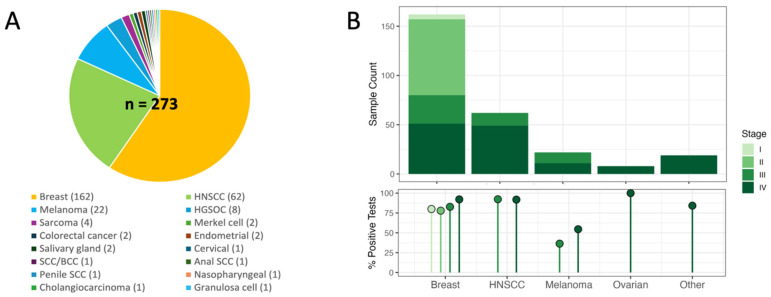
Cohort disease type distribution and baseline pre-treatment ctDNA detection. (**A**) Distribution of patients across all cohorts by cancer type (n = 273). (**B**) Disease stage (**top**) and ctDNA detection rates (**bottom**) for the most common cancer types enrolled. Across the entire cohort, pre-treatment ctDNA was detected in 83% (95%CI 78–87%) of patients (226/273).

A total of 273 patients had pre-treatment plasma samples collected across six cohorts (Tables S1 and S2). Cancer types included breast cancer (n = 162; 59.3%), HNSCC (n = 62, 22.7%), melanoma (n = 22; 8.1%), metastatic recurrent HGSOC (n = 8; 3.0%), and others (n = 19; sarcoma, Merkel cell carcinoma, colorectal cancer, endometrial cancer, salivary gland cancer, cervical cancer, cutaneous SCC/BCC, anal squamous cell carcinoma, penile squamous cell carcinoma, nasopharyngeal cancer, cholangiocarcinoma, and granulosa cell tumor; all stage IV disease) (Figure 1A).

**Figure 2:**
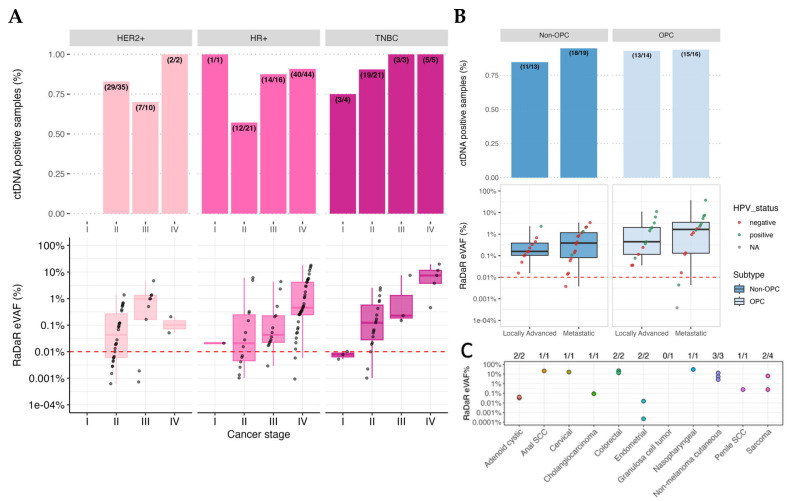
Cancer subtype stratifications of estimated variant allele frequencies. (**A**) Measurement of ctDNA detection and estimated variant allele fraction (eVAF) in breast cancer patients, stratified by receptor subtype and cancer stage. Positive samples below the dotted line had an eVAF of <0.01%. (**B**) HNSCC patients were classified into oropharyngeal (OPC) and non-OPC subtypes. For those that were classified as OPC, HPV status is annotated according to p16 expression, where known. Positive samples below the dotted line had an eVAF of <0.01%. (**C**) Results of ctDNA detection in patients with ‘other’ cancer types.

ctDNA was detected at the pre-treatment timepoint in 83% of patients (226/273; 95%CI: 78–87%) across all stages and cancer types. The detection rate for patients with stage IV metastatic disease (M1) was 89% (110/124; 95%CI 81–93%). When stratified by cancer type, ctDNA detection rates for the most prevalent cancer types in this study were HGSOC (100%; 8/8, all metastatic recurrent; 95%CI 60–100%), breast cancer (stage I 80% [4/5; 95%CI 30–99%]; stage II 78% [60/77; 95%CI 67–86%], stage III 83% [24/29; 95%CI 64–93%], and stage IV 92% [47/51; 95%CI 80–97%]), HNSCC (locally advanced-stage I-IV M0 89% [24/27; 95%CI 70–97%], and metastatic M1 94% [33/35; 95%CI 79–99%]), melanoma (stage III 36% [4/11; 95%CI 12–68%], stage IV 54% [6/11; 95%CI 25–82%]), and other tumor types (84% [16/19]; 95%CI 60–96%) (Figure 1B). When stratifying by breast cancer receptor subtypes (hormone receptor and HER2), ctDNA detection was 82% (67/82; 95%CI 71–89%) in HR+/HER2−, 81% (38/47; 95%CI 66–90%) in HER2+, and 91% (30/33; 95%CI 74–98%) in triple-negative disease (Figure 2A). In HNSCC, detection rates across the different primary sites of disease were as follows: oropharyngeal (94%, 32/34; 95% CI 79–99%), oral cavity (85%, 11/13; 95%CI 54–97%), larynx (89%, 8/9; 95%CI 51–99%), hypopharynx (100%, 4/4; 95%CI 40–100%), and other sites (100%, 2/2; 95%CI 20–100%) (Figure 2B).

**Figure 3:**
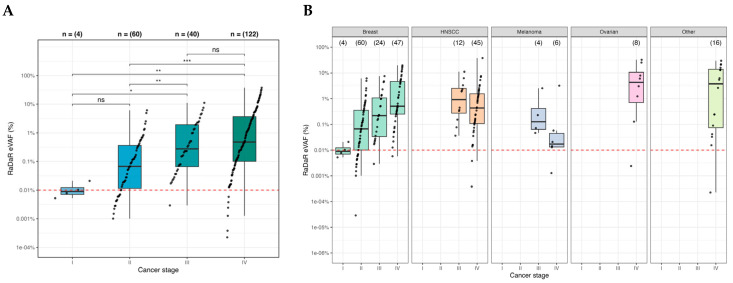
Estimated variant allele frequencies from patients with a positive ctDNA test. (**A**) Median eVAF across all patients with a positive ctDNA result, by cancer stage. Statistical significance was assessed by the Kruskal-Wallis test. Median eVAF observed across all patients was 0.25% (range: 0.000029–37.7%) and increased with cancer stage (pairwise comparisons held statistical significance annotations as follows: ns *p* > 0.05, * *p* ≤ 0.05, ** *p* ≤ 0.01, *** *p* ≤ 0.001). Overall, 11% (24/226) of positive samples had an eVAF of <0.01%. (**B**) Individual eVAF measurements were reported, stratified by cancer type and their associated stage (Table S2). The red dashed line separates samples with ctDNA detected at levels greater than or less than 0.01% eVAF.

Next, relative ctDNA detection levels were calculated using the estimated variant allele fraction (eVAFs) in ctDNA-positive samples. The median eVAF observed across ctDNA-positive patients was 0.25% (range: 0.000029–37.7%). Overall, eVAF increased with tumor stage (Figure 3A). A total of 11% (24/226) of positive samples had an eVAF of <0.01%, supporting the importance of assay sensitivity for accurate characterization of ctDNA status. The median eVAF varied by tumor type and stage: metastatic recurrent HGSOC (stage IV 4.5% [n = 8]), breast cancer (stage I 0.009% [n = 4], stage II 0.07% [n = 60], stage III 0.22% [n = 24], stage IV 0.50% [n = 47]), HNSCC (locally advanced-stage I–IV M0 disease 0.28% [n = 24], metastatic M1 disease 0.95% [n = 33]), and melanoma (stage III 0.15% [n = 4] and stage IV 0.018% [n = 6]) (Figure 3B). A trend towards increasing eVAF with stage was observed in breast cancer HR+ and TNBC disease when stratified by receptor status subtypes (Figure 2A). When HNSCC oropharyngeal (OPC) patients were considered separately from non-OPC patients, ctDNA-positive OPC samples exhibited higher eVAF than ctDNA-positive non-OPC samples overall (*p* = 0.004), where the median eVAF (%) for OPC was 1.5% (range: 0.0004–27%), and for non-OPC, it was 0.23% (range: 0.0034–3.41%) (Figure 2B). Among patients with OPC, those who were HPV+ had higher eVAFs than those with HPV− disease (*p* = 0.0004, Wilcoxon’s test). A wide range of eVAFs were observed among the “other” tumor types with positive ctDNA detection (n = 16/19) (Figure 2C).

## 3. Discussion

In this study, ctDNA detection rates at a single pre-treatment time point were analyzed across six prospective clinical cohorts, including 273 patients with solid tumor malignancies spanning 16 cancer types. All patients were recruited at a single institution, with the majority having samples collected and processed using the same protocols (Table S1). ctDNA was analyzed using the RaDaR^®^ tumor-informed ctDNA assay, enabling standardized detection and quantification across diverse clinical contexts.

The pre-treatment pan-cancer ctDNA detection rate was high, at 83% (226/273, Figure 1), though detection rates varied by cancer type, stage, and other clinical characteristics. For example, detection rates were 100% (95% CI 60–100%) in metastatic recurrent HGSOC (n = 8/8), but only 54% (95% CI 25–82%) in stage IV melanoma (n = 6/11). Other studies have also reported lower ctDNA detection rates in melanoma, even in stage IV disease [20,21], although some variability does exist. Across the entire patient cohort, however, eVAF increased with disease stage (Figure 2), including trends within the breast cancer and HNSCC subtypes. eVAF in OPC patients was statistically higher relative to non-OPC patients. Of note, and perhaps unexpected given the selection for patients with known active disease, many of which had advanced disease), a relatively high proportion (11%) of positive samples had ctDNA detected at levels below 0.01% eVAF. This adds to a growing body of evidence highlighting the importance of assay sensitivity to correctly characterize and quantify ctDNA in patient samples. The described eVAF distributions may help identify scenarios in which assays with greater or lesser sensitivity are most appropriate and may also provide insight into settings where ctDNA levels are sufficient to support standard approaches to genomic profiling or mutation detection.

While some variation in ctDNA detection may be attributable to factors such as asymmetric distributions of cancer stage within subgroups, biological mechanisms are expected to account for at least some of these important findings. ctDNA detection is known to be influenced by mechanisms of cfDNA and ctDNA release (e.g., apoptosis, necrosis, pyroptosis, extracellular vesicle release) and degradation (e.g., by nucleases) and has been associated with other clinicopathological features, including proliferation status and tumor grade [15,22,23,24,25,26]. ctDNA detection can be affected by factors such as overall tumor burden and anatomic location of the disease [4,5,6], as well as by cancer treatments [7,8,9,10]. Even within a single cancer type, variation was observed, including higher eVAF levels in samples from patients with HPV+ OPC samples compared to HPV− OPC (Figure 3B). Factors such as tumor-infiltrating lymphocytes (TILs) [27] and inflammatory mediators and cytokines [28] may influence ctDNA release, but their interactions remain largely theoretical. Despite the knowledge accumulated to date, the mechanisms and clinical implications of ctDNA biology remain active areas of investigation. The variability in ctDNA shedding, as well as the analytical sensitivity/specificity of the assay, should be taken into consideration when assessing its specific clinical application.

Overall, this study provides a broad survey of ctDNA detection and high-sensitivity quantification in a cohort of patients sampled and measured in a standardized way. The analysis does have several limitations. First, cancer type distribution was not even, with a relative abundance of patients with breast cancer, and few patients with some less common tumor types. In addition, within cancer types, subgroup analyses may be confounded by the non-uniform distribution of disease stages. Detailed clinical data were not assessed in this analysis, which focused on reporting detection rates. Associations with standardized outcomes, such as relapse-free survival and overall survival, have been separately described in relevant cohorts ([29,30,31,32,33]; NCT02644369). In addition, despite efforts to harmonize methodologies across cohorts, differences existed between BIO2 and the remaining studies performed under LIBERATE, including the use of EDTA rather than Streck blood collection tubes. Further, these results reflect the detection performance of a single commercial ctDNA assay based on SNV detection, which required successful generation of a patient-specific panel based on tumor WES. Other approaches, including WGS-based methods with lower limits of detection, or tumor-agnostic approaches employing fragmentomics [34] or methylation [35], may yield different rates of detection or permit analysis of samples where a tumor is unavailable. Lastly, the single-institution design of this study may limit generalizability based on the processing techniques or patient populations enrolled. Notwithstanding these factors, this cohort represents one of the few reported ctDNA studies providing a landscape of variant detection across multiple cancers and clinical settings.

## 4. Materials and Methods

### 4.1. Study Cohorts and Plasma Collection

Six patient cohorts were included in this study, encompassing a total of 273 patients (Figure 1A). Five of these cohorts (TRACER [29], ACUITI [30], Pre-MERIDIAN [31], SAMBA [32], and MASST [33]) had peripheral blood plasma and tissue collected using the same methodology, as part of the institutional liquid biopsy collection study entitled ‘Liquid Biopsy Evaluation and Repository Development at Princess Margaret’ (LIBERATE; NCT03702309). Patients in the BIO2 cohort had blood and tissue collected using similar methods, but under a separate study (NCT02644369). Cohorts are summarized briefly here and were all conducted with institutional Research Ethics Board (REB) approval at the University Health Network (Princess Margaret Cancer Centre, Toronto, Canada) (Table S1).

TRACER was a longitudinal study of patients with early breast cancer, enrolled prior to neoadjuvant therapy, to assess treatment-related ctDNA dynamics (n = 114; REB #17-5962). ACUITI was a prospective longitudinal study of patients with metastatic hormone receptor-positive (HR+), HER2-negative breast cancer treated with standard endocrine therapy and CDK4/6 inhibitors in the first- and second-line setting for correlative analysis, including ctDNA assessment (n = 43; REB #18-6192). MASST was a prospective observational study of patients with recurrent metastatic squamous cell carcinoma of the head and neck (HNSCC), esophagus, or anal canal, starting first-line platinum-based chemotherapy or any line of immunotherapy treatment; only HNSCC patients from MASST were included in this study (n = 40; REB #18-5407). Pre-MERIDIAN was a prospective observational study of patients with high-risk HNSCC, treated with curative intent therapy for biomarker evaluation, including ctDNA detection (n = 17; REB #20-5804). SAMBA was a prospective longitudinal observational study of high-risk melanoma patients treated with curative intent for correlative assessment, including ctDNA monitoring (n = 18; REB #19-5694). BIO2 is a bio-banking sub-study of the INSPIRE clinical trial (NCT02644369; REB #15-9828), which was a phase 2 investigator-initiated interventional study of pembrolizumab in patients with advanced solid tumors (n = 41). BIO2 cohorts included patients with HNSCC, triple-negative breast cancer (TNBC), high-grade serous ovarian cancer (HGSOC), metastatic melanoma, and mixed advanced solid tumors. Across all studies, patients with HNSCC were further classified into those with locally advanced-stage I-IV M0 non-metastatic disease (M0) or overt metastatic disease (M1). For patients with HNSCC of oropharyngeal (OPC) primary, HPV status was reported from p16 expression data that were available through clinical documentation.

### 4.2. Tissue, Blood, and ctDNA Analyses

WES, ctDNA panel design, cell-free DNA extraction, and processing of sequencing data were completed as previously described [29,36,37]. Tumor-only WES was used to identify somatic variants for cohorts enrolled under the LIBERATE platform (FFPE), and tumor-normal WES was used for BIO2 (fresh frozen or FFPE samples). For samples sequenced under LIBERATE, tissue was sent to NeoGenomics Laboratories, Inc. (Durham, NC, USA), where DNA was extracted using Maxwell RSC DNA FFPE kits. Extracted DNA was processed with the KAPA Hyper Prep Kit and indexed uniquely. Each library proceeded to exome enrichment and was analyzed on a fragment analyzer and quantified using the Quant-iT dsDNA High-Sensitivity assay. Sequencing was performed on the HiSeq4000 platform (Illumina, San Diego, CA, USA). For samples analyzed in the BIO2 cohort, DNA was extracted from tumor biopsies and then sequenced with Illumina at the Princess Margaret Genomic Centre and the Princess Margaret-Ontario Institute of Cancer Research Translational Genomics Laboratory (PM-OICR TGL) in Toronto, Canada [37]. Exonic regions were enriched using Agilent SureSelectXT (Santa Clara, CA, USA) target enrichment and hybridization to Agilent SureSelect Human All Exon V5 + UTRs baits. Pooled libraries were sequenced using either HiSeq2000 or HiSeq2500. Paired-end 125 bp reads were generated to target a median coverage of 250x for tumor samples and 50x for control blood DNA. WES data were sent for processing through RaDaR’s pipeline for variant identification and prioritization and design of personalized MRD assays.

Venous blood from the study’s participants was collected using standard phlebotomy techniques prior to the initiation of the protocol-specified line of therapy in each respective study. For cohorts enrolled under LIBERATE, Streck tubes were used for plasma collection and prepared as soon as possible after collection and within 7 days, with an initial centrifugation at 1600× *g* for 10 min and a second centrifugation of plasma aliquots at 20,000× *g* for 10 min. The buffy coat layer was separated during blood processing and stored at −80 °C. DNA was extracted from 200 µL of buffy coat (leukocytes) using the QIAamp DNA Blood Mini Kit (Qiagen, Redwood City, CA, USA) and circulating cell-free DNA, extracted using the QIAamp Circulating Nucleic Acid Kit (Qiagen). For BIO2, EDTA tubes were employed for plasma collection with an initial centrifugation at 1900× *g* for 10 min and a second centrifugation of plasma aliquots at 16,000× *g* for 10 min. Peripheral blood mononuclear cells (PBMCs) were isolated by Ficoll gradient centrifugation, and snap-frozen PBMC pellets were stored at −80 °C. cfDNA was purified from plasma using the QIAamp Circulating Nucleic Acid Kit (Qiagen).

RaDaR is a personalized ctDNA assay that utilizes multiplex PCR and targeted NGS. Somatic variants identified in the tumor tissue by WES were prioritized using proprietary algorithms to build a patient-specific primer panel of up to 48 primer pairs, capturing at least one somatic variant. The personalized primer panel was combined with a fixed primer panel of 21 common population-specific SNPs for quality control purposes during the NGS testing. An aliquot of tumor DNA from FFPE tissue was used during panel QC to confirm the accuracy and performance of each personalized assay. Following primer panel qualification, assays were performed on cfDNA from plasma aliquots alongside a buffy coat DNA control sample, which was used for identification and removal of germline variants, the removal of variants due to clonal hematopoiesis of indeterminate potential (CHIP), and as a positive amplification control. Multiplex PCR was performed with input concentrations, as measured by droplet digital PCR. RaDaR libraries were sequenced using the NovaSeq 6000 system (Illumina Inc., San Diego, CA, USA), and sequencing data were analyzed in a multi-step process: fastq files were demultiplexed using *bcl2fastq (version 2.20)*, reads were then aligned using the *bwa mem (version 0.7.17)* alignment software, and processed using proprietary software to identify primer pairs and count mutant and reference bases. Samples were ultimately classified as ctDNA positive when the cumulative statistical score was above the pre-set threshold defined during the assay’s analytical development.

Differences in ctDNA detection rates were analyzed using the Wilcoxon rank-sum, chi-square, or Fisher’s exact test, or Kruskal-Wallis test, as appropriate. When describing ctDNA detection rates within different groups, 95% confidence intervals incorporating continuity correction were provided. As this was an exploratory and hypothesis-generating study, correction for multiple testing was not applied for all comparisons. All statistical analyses were carried out in R (version 4.2.1), using two-sided tests with an alpha level of significance of <0.05.

## 5. Conclusions

In summary, this study provides a landscape of pan-cancer ctDNA detection and quantification using a standardized assay with high analytical sensitivity. Overall detection rates were high, with approximately 10% of ctDNA positive samples having ctDNA detected at an eVAF of <0.01%. These results provide a useful reference for ongoing efforts to develop clinical applications for ctDNA use across various cancer types and clinical contexts, including efforts to individualize cancer therapy, monitor its effects, or provide surveillance following completion of treatment.

## Data Availability

The original contributions presented in this study are included in the article/Supplementary Materials. Further inquiries can be directed to the corresponding author.

## Supplementary Materials

The following supporting information can be downloaded at: https://www.mdpi.com/article/10.3390/ijms27093979/s1.
